# “Clinical comparison of bond failure rate between two types of mandibular canine-canine bonded orthodontic retainers- a randomized clinical trial”

**DOI:** 10.1186/s12903-020-01167-7

**Published:** 2020-06-29

**Authors:** Nasreen Iqbal Nagani, Imtiaz Ahmed, Faiqa Tanveer, Hafiza Marium Khursheed, Waqas Ahmed Farooqui

**Affiliations:** 1 Department Of Orthodontics, Dr. Ishrat-Ul-Ebad Khan Institute Of Oral Health Sciences (DIKIOHS), Dow University Of Health Sciences, Karachi, Pakistan; 2 Present Address: Adam Plaza, flat no 103, opp: New Town Masjid, Gurumandir, Karachi, Pakistan; 3 Department of Research, School of Public Health, Dow University Of Health Sciences Karachi, Karachi, Pakistan

**Keywords:** Fixed retention, Fiber reinforced composite, Multistranded wire, Bond failure

## Abstract

**Background:**

Bonded retainers are widely used as they are esthetically pleasing, easily acceptable, provide greater stability, compliance free and causes no soft tissue irritation and speech problems. Though, fracture and bond failure are their shortcomings. Therefore, the objectives of this study were to evaluate the number of bond failures and type of failure pattern between two types of mandibular canine-canine bonded retainers.

**Methods:**

Total 60 subjects were recruited initially and were assessed for eligibility, out of which 6 were excluded and 2 were lost to follow up. They were randomly divided into two groups. Fiber reinforced composite (FRC) retainers were inserted in group 1 subjects while group 2 subjects received multistranded stainless steel (MSW) retainers. The subjects were recalled after every 3 months over a period of 1 year. Bond failure rate and failure pattern based on adhesive remnant index were evaluated at each visit. The bond failure rate and failure pattern were compared between the two retainers by using Chi-square test.

**Results:**

The bond failure rates were 42.94% for FRC retainer and 31.41% for MSW retainer. Hence, total number of bond failures in both retainers were 37.17%. The difference of bond failure between two groups was statistically significant (*p* = 0.012). Type “0” failure pattern was detected commonly with both types of retainers (*p* <  0.001).

**Conclusion:**

Our findings indicate that multistranded stainless steel wire retainer is a superior option to be used for fixed lingual retention in mandibular arch as it exhibited lower bond failure as compared to fiber reinforced composite retainer. Adhesive failure is the most common type of bond failure observed with both types of fixed retainers.

**Trial registration:**

ID NCT03881813 (https://clinicaltrials.gov/); March 19, 2019, retrospective registration.

## Background

Retention is an integral phase after the completion of orthodontic treatment [1]. It is the obligatory stage that enables the dentition to adapt the newly attained position and to maintain the stability and alignment of the teeth which otherwise will revert back to their pretreatment position resulting in relapse. Post retention orthodontic treatment records reveal loss of stability and alignment specifically in the mandibular anterior region [2]. Therefore permanent retention is highly recommended to ensure the stability and to maintain the long term effects of the dentition achieved by the treatment. These consequences can be accomplished by a fixed lingual retainer inserted for an optimum time interval [3, 4].

Zachrisson was the first one who boosted the usage of multistranded stainless steel wire (MSW) as fixed lingual retainers instead of round SS wire [5]. Fixed lingual retainers are specifically inserted in cases of generalized spacing, midline diastemas or to maintain implant/ pontic spaces. However with the passage of time, these are preferred usually in all cases as they offer numerous benefits for instance they are esthetically pleasing, easily acceptable, provide greater stability, compliance free and causes no soft tissue irritation and speech problems [6]. Although, they are technique sensitive and time consuming. Fracture and bond failure are also reported [7, 8].

Bond failure may be of cohesive or adhesive type. Cohesive failure is the type of failure at wire adhesive interface which occurs due to inadequate composite resin whereas, adhesive failure which is between adhesive and enamel surface results from movement of wire during bonding or moisture contamination [8]. The failure rates ranging from 10.3 to 47.0% have been reported [9]. Various factors that determine the survival rate of retainers are site of placement, type of material used, total number of teeth bonded and type of composite resin used [10, 11].

Multistranded stainless steel wire (MSW) of varying diameter is frequently used as fixed lingual retainer [12]. More recently fiber reinforced composite (FRC) materials found their way into orthodontics [13]. These are based on fiber laminate technology which involve the use of closely adapted layers of reinforcement fibers made up of carbon, polyaramid, polyethylene and glass held in place by a thin resin bonding layer [14–16]. They adhere to the surfaces of the teeth chemically [17] and diffuse the forces to the glass fibers hence reinforcing the resistance offered by the bonding agent [18]. They offer adequate strength due to the incorporation of glass fibers into the composite resin ultimately results in better durability [19, 20]. Their esthetic nature and biocompatibility are remarkable features [2, 21]. They are conformed easily along the lingual surfaces of teeth and are lighter in weight [22, 23]. Likewise their complete detachment occurs rarely and are fixed easily [6]. Nevertheless they allow restricted physiological tooth movement as they create a stiff splint which may lead to their high bond failure [9]. To the best of our knowledge few studies have been conducted on these latest materials which revealed that the selection of the best type of fixed lingual retainer still remains a subjective issue hence, their reliability is yet questionable [24, 25]. As, these studies, and their synthesis, cannot provide reliable evidence in this field, therefore this trial was conducted to fill this gap.

Therefore, the objectives of this study were to evaluate the number of bond failures and type of failure pattern based on adhesive remnant index between two types of mandibular canine-canine bonded retainers.

## Methods

This trial was approved by the Institutional Review Board of Dow University of Health Sciences (Ref:IRB-941/DUHS/Approval/2017/162) and was registered under the protocol ID NCT03881813 (https://clinicaltrials.gov/), according to the CONSORT statement of the updated guidelines for reporting randomized clinical trials.

### Study design and settings

It was a parallel-group randomized clinical trial, multicenter study with a 1:1 allocation ratio, conducted at the department of Orthodontics, Dr. Ishrat Ul Ebad Khan Institute Of Oral Health Sciences (DIKIOHS) and Dow Dental College (DDC) in Dow University of Health Sciences (DUHS) for the period of 17 months.

### Sample size calculation

**Fig. 1:**
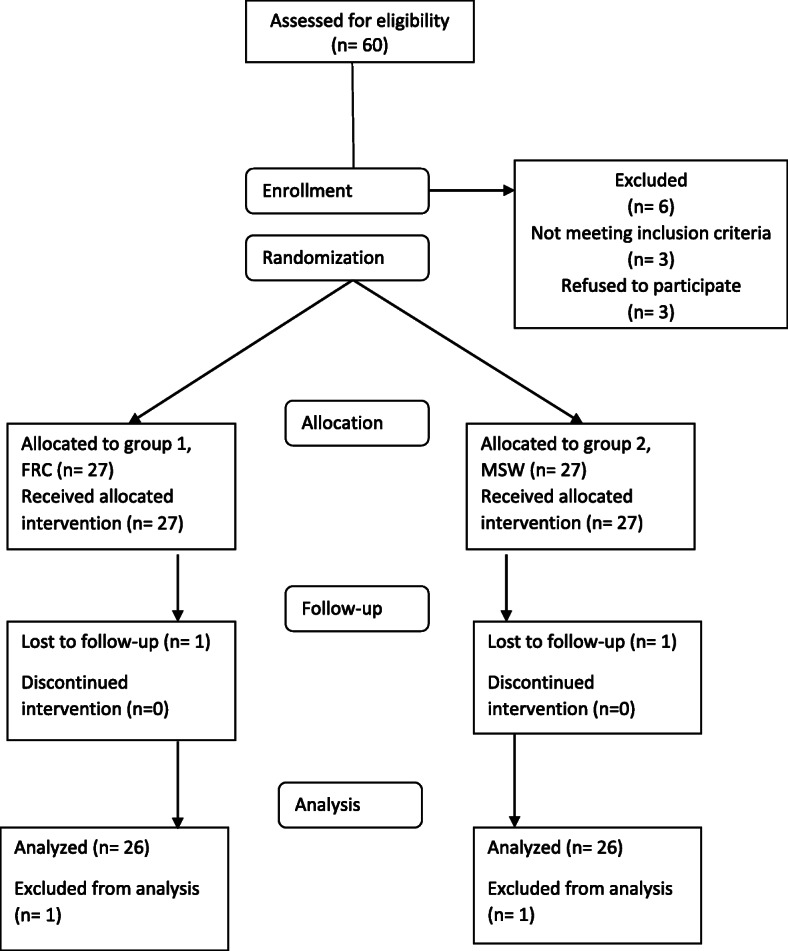
Patient flow chart during the trial

The sample size of 56 subjects was calculated using PASS version 11, based on two sample proportion with 95% confidence of interval and 80% power of test, bond failure [26] in flexible spiral wire (17.73%) and fiber reinforced composite (11.25%) with estimated population size of 60 patients in 6 months. Keeping in view, the inclusion of dropout rate, total 60 subjects were recruited. (Fig. 1) Purposive sampling technique was followed.

### Patients recruitment and follow-up

The subjects were randomly divided through computer generated software (Microsoft Excel) into two groups. Retainers were inserted by the principal investigator. Non extraction cases below the age of 45 with moderate crowding and normal facial pattern who agreed to visit after every 3 months for a follow-up of 1 year and were treated with MBT fixed appliance were included while subjects with congenitally missing anterior teeth and poor oral hygiene were excluded.

After selection of subjects debonding, deep scaling and curettage was performed for each individual. Prior to the bonding of fixed retainer, all composite remnants were removed and enamel surfaces were cleaned with tungsten carbide bur in slow speed hand piece. Two types of fixed lingual retainers were bonded in the mandibular arch from canine-canine to all the six anterior teeth by a single operator. Group 1 subjects received fiber reinforced composite retainers (INOD, U.P. Fiber Splint, 2 mm) while group 2 (control group) subjects received multistranded stainless steel wire retainers (All Star Orthodontics, 0.0175 in.).

### Fiber reinforced composite (FRC) group

**Fig. 2:**
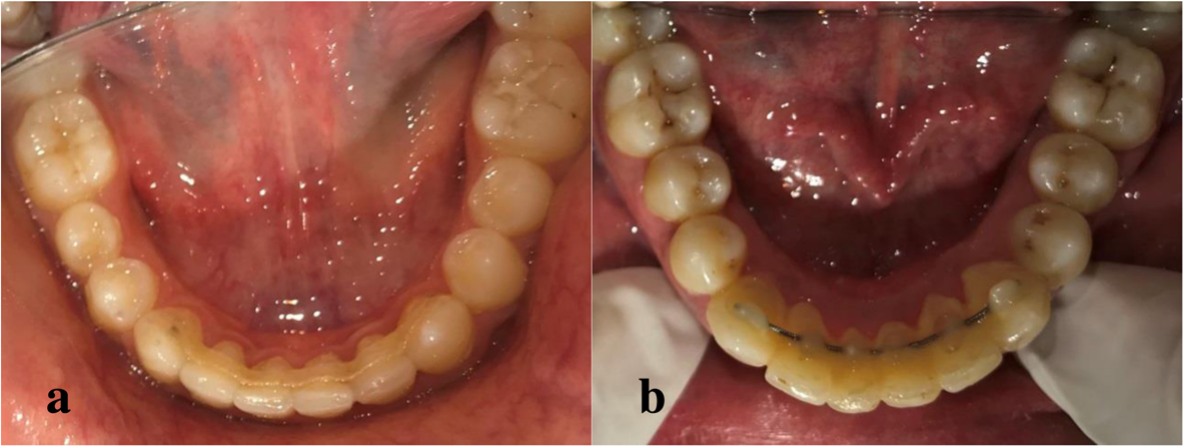
**a**: FRC retainer bonded on the lingual surfaces of mandibular anterior teeth. **b**: MSW retainer bonded on the lingual surfaces of mandibular anterior teeth

After isolation of mandibular anterior teeth, dental floss was used for canine-canine distance measurement. Adequate length of ribbon fiber was cut by scalpel blade. It was pretreated with adhesive primer (3 M ESPE). Lingual surfaces of anterior teeth were etched with 37% phosphoric acid gel (Meta Biomed) for 30 s, were washed adequately and air dried. Then adhesive primer (3 M ESPE) was applied with applicator brush. Eventually each tooth was light cured with a light emitting diode (Otholux; 3 M) for 15 s which was followed by the application of flowable composite resin (3 M ESPE). Then fiber ribbon was adapted to the lingual surfaces of six anterior teeth with plastic instrument, surplus composite was cleared and each tooth was light cured for 15 s. Strict oral hygiene instructions were conveyed to all the subjects. (Fig. 2a).

### Multistranded stainless steel wire (MSW) group

In MSW group, same isolation and bonding protocols were followed. (Fig. 2b).

### Primary outcome measure (failure rate) and secondary outcome measure (ARI)

After insertion of retainers, the patients were recalled after every 3 months for a period of 12 months. On each visit, bond failure rate and pattern of failure via adhesive remnant index (0, no retained resin on enamel surface; 1, < 50% retained resin on enamel surface; 2, > 50% retained resin on enamel surface and 3, all resin retained on enamel surface) were observed [27]. In case if detachment of retainer was noticed during the visit, the subject was allowed to approach immediately for repair and bond failure was recorded in the upcoming visit.

### Statistical analysis

Total 60 subjects were recruited, out of which 6 were excluded. Out of 54 subjects, two dropped out during the study; hence data was analyzed on 52 subjects using Statistical Package for Social Sciences (SPSS) Version 21. Cross tabulation was designed to detect the type of failure pattern. The bond failure rate and failure pattern were compared between the two retainers by using Chi-square test with *p*-value < 0.05 as significant.

## Results

Total 60 subjects were recruited, 6 were excluded and 2 were lost to follow up so data was analyzed on 52 subjects for 12 months. The mean age of subjects were 21.5 ± 3.6 with the range of 14–30 years. Out of total subjects, 8 (15.4%) were males while 44 (84.6%) were females. Out of 52 cases, 38 (73.1%) were class I malocclusion treated while 14 (26.9%) were class II treated cases. There were no significant differences in the baseline characteristics i.e. age, gender and type of malocclusion between the two groups. (Table 1).

**Table 1:** Comparison of baseline characteristics between two retainers

Baseline characteristics	FRC	MSW	***p***-value *
**Age**			
mean ± SD	20.88 ± 3.45	22.15 ± 3.68	0.206
**Gender**			
Male n(%)	4 (50.0)	4 (50.0)	1.000
Female n(%)	22 (50.0)	22 (50.0)	
**Malocclusion**			
Class I	21 (55.3)	17 (44.7)	0.211
Class II	5 (35.7)	9 (64.3)	

*FRC* Fiber reinforced composite, *MSW* Multistranded stainless steel wire

*p-values calculated using Independent t-test and Chi-square test

The bond failure rates were 42.94% for FRC group and 31.41% for MSW group. Hence, total number of bond failures in both groups were 37.17%. The difference of bond failure between two groups was statistically significant with *p* = 0.012. (Table 2).

**Table 2:** Comparison of bond failure between FRC and MSW retainers

Type of retainer	No. of subjects	No. of teeth bonded	Frequency of bond failure (%)	Significance (***P***-value)*
FRC	26	156	67 (42.94%)	0.012
MSW	26	156	49 (31.41%)	
Total	52	312	116 (37.17%)	

*FRC* Fiber reinforced composite, *MSW* Multistranded stainless steel wire

*Significant at 0.05 (Chi-square test)

When failure pattern was compared between the two retainers (Table 3), the frequency of type “0” failure pattern was 45 (62.5%) in FRC group and 27 (37.5%) in MSW group with total frequency of 72 in both groups. The frequency of type “1” pattern in FRC group was 18 (85.7%) and 3 (14.3%) in MSW group. Total frequency in both retainers was observed to be 21. Similarly, frequency of type “2” pattern was 3 (20%) in FRC and 12 (80%) in MSW group. Total frequency was 15 in both groups. The frequency of type “3” failure pattern was 1 (12.5%) in FRC and 7 (87.5%) in MSW group. Total frequency in both groups was 8. The difference of failure pattern was statistically significant (*p* <  0.001).

**Table 3:** Comparison of failure patterns (ARI) between FRC and MSW groups

Failure pattern (ARI)	FRC	MSW	Total	Significance (***P***-value)*
**0**	45 (62.5%)	27 (37.5%)	72	< 0.001
**1**	18 (85.7%)	3 (14.3%)	21	
**2**	3 (20%)	12 (80%)	15	
**3**	1 (12.5%)	7 (87.5%)	8	

Note: (calculated percentages are row percentages)

*ARI* adhesive remnant index, *FRC* Fiber reinforced composite, *MSW* Multistranded stainless steel wire

*Significant at 0.05 (Chi-square test)

## Discussion

In this study the difference of bond failures between the two retainers were clinically and statistically significant (*P* < 0.05). The results of our study showed that there is greater and significant bond failure observed with FRC retainers as compared to MSW retainers over a follow-up of 1 year.

The results of our study are in accordance with the study conducted by Rose and Tacken [13, 28]. They reported that multistranded stainless steel retainers are superior to the fiber reinforced in terms of reliability and bond failure. Similarly in vitro study conducted by Foek et al. reported that stainless steel wire retainers showed significantly higher bond strength as compared to fiber reinforced retainers [19]. The possible reasons for these findings are increased rigidity and strain levels of FRC retainer as compared to MSW retainer which results in increased masticatory load and bond failure [29]. Furthermore, FRC due to increased diameter of 2 mm as compared to MSW, covers greater surface area of teeth resulting in increased bond failure. It has also been reported by Scribante et al. that increased bond failure in FRC occurs due to weak interface between fiber and organic matrix as a result of intraoral hydrolysis and degradation [16] whereas, braided surface of MSW shows increased mechanical retention leads to decreased failure rate [30]. Sfondrini et al. compared the clinical reliability and bond failure between fiber reinforced composite and spiral wire retainers in the mandibular anterior teeth over 12 months period [26]. The current study is also conducted on these fixed retainers on mandibular teeth for 12 months. The number and rate of bond failure and cause were evaluated for two types of retainers. Bond failure rate was higher for multistranded wire retainer as compared to fiber reinforced composite retainer but the results were insignificant.

The present study also evaluated the type of failure pattern based on adhesive remnant index. The results showed that all types of failure patterns were observed but type “0” was found frequently in both types of fixed retainers while type “3” pattern was rarely found. These finding are comparable with the findings of previous studies [31, 32] which also reported similar results. Possible causes of failure at this point is the contamination of enamel surface during bonding and poor moisture control [31]. Other causes are insufficient or over etching of enamel surface, inadequate drying and faulty bonding procedure [32]. Type “0” failure pattern indicates that there is no resin left on the tooth surface after bond failure occurred, which means adhesive bond failure (enamel-adhesive junction) was detected commonly. In these instances, enamel surface was clear. When type “1” pattern was detected, partial adhesive remnants were present on tooth surface as compared to retainer surface which indicates that < 50% adhesive is found on tooth surface. When type “2” failure pattern was detected, adhesive remnants on tooth surface were greater as compared to retainer surface which indicates that > 50% adhesive is left on tooth surface. Type “3” pattern was detected least commonly specially, in FRC group, when no remnants were present on retainer surface. It is cohesive bond failure (adhesive-retainer junction) which indicates that all adhesive is present on tooth surface.

The strength of this study includes, that this study is a randomized clinical trial and allocation of subjects through randomization minimizes the risk of selection bias. Although blinding was not possible for the interventions given as they were visible on the surfaces of the teeth, blinding was done for the outcome assessment which minimizes the observation and detection biases. Moreover, it is a multicenter study which involves the recruitment of subjects from different areas, thus sample of subjects is representative and the results are assumed to be more reliable and generalized. However, limitations of the study include difficulty in controlling certain factors that might be accountable for the detachment or breakage of the retainers such as biting on the hard food, object or brushing aggressively with a stiff brush and masticatory forces. Retainers were repaired as soon as the subject reported their breakage but at times, the subjects were incapable to make out the detachments or breakages during the visits and were detected and repaired by the clinician on the follow-up visits. Furthermore, number of broken retainers were not recorded in the same patients i.e. clustering effects were not evaluated.

It is recommended to evaluate other types of fiber reinforced composites such as nanofilled in future. Further studies to assess the effects of the fixed retainers on periodontal health status and arch width changes in the post retention phase should be considered.

## Conclusion

Our findings indicate that multistranded stainless steel wire retainer is a superior option to be used for fixed lingual retention in mandibular arch as it exhibited lower bond failure as compared to fiber reinforced composite retainer. Different types of failure patterns have been observed but adhesive failure is the most common type of bond failure observed with both groups of fixed lingual retainers.

## Data Availability

The datasets used and/or analyzed during the current study are available from the corresponding author on reasonable request.

## Abbreviations

FRC: Fiber reinforced composite
MSW: Mutistranded stainless steel wire
SPSS: Statistical package for social sciences
